# Endoscopic submucosal dissection using a novel therapeutic thin gastroscope for a locally recurrent rectal tumor after endoluminal rectal surgery

**DOI:** 10.1055/a-2174-5398

**Published:** 2023-10-06

**Authors:** Yusaku Takatori, Noriko Matsuura, Atsushi Nakayama, Motohiko Kato, Naohisa Yahagi

**Affiliations:** 1 Division of Research and Development for Minimally Invasive Treatment, Cancer Center, Keio University School of Medicine, Tokyo, Japan; 2 Center for Diagnostic and Therapeutic Endoscopy, Keio University School of Medicine, Tokyo, Japan

The lower rectum is the part of the gastrointestinal tract that gains most benefit from minimally invasive treatment such as endoscopic resection, because invasive surgery results in such a significant decrease in quality of life 1 . However, endoscopic resection in the lower rectum is sometimes technically difficult due to the narrow lumen and steep angle of the rectal wall. Here, we report the first description of endoscopic resection in the lower rectum using a novel thin therapeutic gastroscope.

A 70-year-old woman was referred to our hospital for treatment of a recurrent rectal tumor after endoluminal rectal surgery, which was performed at a different hospital. The lesion was located on the post-surgery scar at the posterior wall of the lower rectum ( Fig. 1 ). Maneuverability of the endoscope was limited because the anal side of the lesion was within a confined space adjacent to the anal canal. Moreover, it was difficult to approach the lesion even by retroflexion because the gastroscope faced perpendicularly to the steep rectal wall. Therefore, we performed endoscopic submucosal dissection using a novel therapeutic thin gastroscope (EG-840TP; Fujifilm Corp., Tokyo, Japan), which has a thinner diameter (7.9 mm) and a wider range of down angles (160°degree) than existing therapeutic gastroscopes ( Fig. 2 ).

**Fig. 1:**
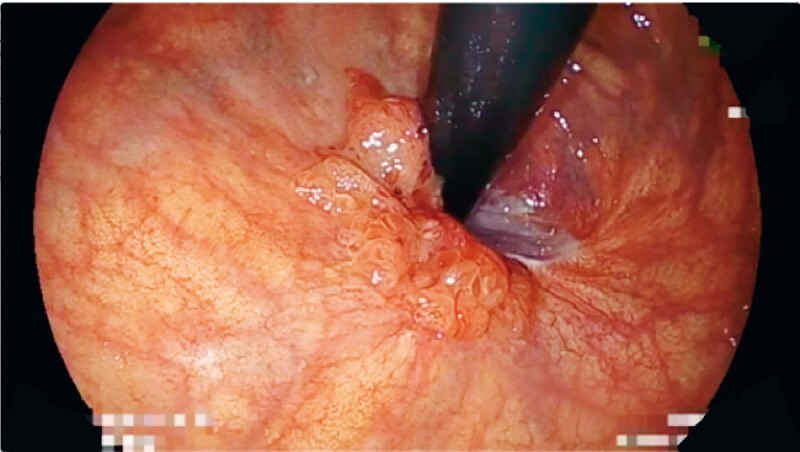
The target lesion was located on the post-surgery scar at the posterior wall of the lower rectum.

**Fig. 2:**
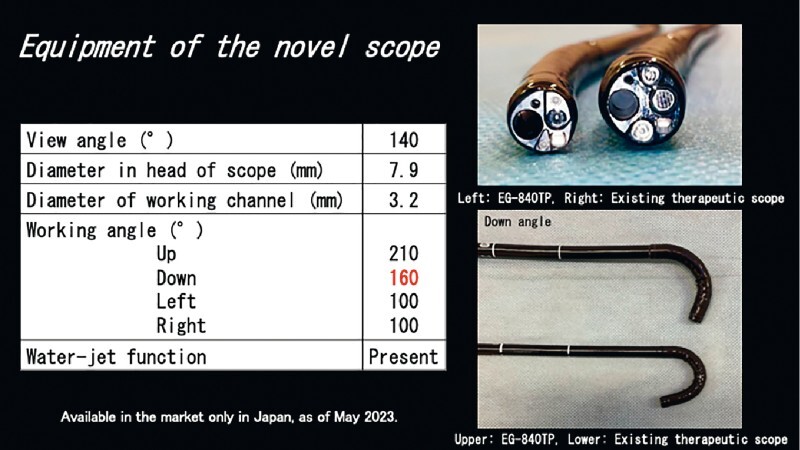
Details of the novel therapeutic thin gastroscope.

The procedure was carried out with the scope in the straight position ( Video 1 ). The thin diameter of the scope was very useful even in a confined space and made it easy to enter the submucosal layer. The wider range of down angles enabled the endoscopic knife to approach at a precise depth in the submucosal layer. Finally, the lesion was resected en bloc without any adverse events.

**Video 1** Endoscopic submucosal dissection of the target lesion using a novel therapeutic thin gastroscope.


This case suggests that the novel thin gastroscope may be an option for endoscopic resection in lower gastrointestinal tumors.

Endoscopy_UCTN_Code_TTT_1AQ_2AC

## Notes

Endoscopy E-Videos

https://eref.thieme.de/e-videos
*E-Videos* is an open access online section of the journal *Endoscopy* , reporting on interesting cases and new techniques in gastroenterological endoscopy. All papers include a high-quality video and are published with a Creative Commons CC-BY license. Endoscopy E-Videos qualify for HINARI discounts and waivers and eligibility is automatically checked during the submission process. We grant 100% waivers to articles whose corresponding authors are based in Group A countries and 50% waivers to those who are based in Group B countries as classified by Research4Life (see: https://www.research4life.org/access/eligibility/ ). This section has its own submission website at https://mc.manuscriptcentral.com/e-videos
